# Irisin relaxes rat thoracic aorta through inhibiting signaling pathways implicating protein kinase C

**DOI:** 10.3906/sag-2105-113

**Published:** 2021-11-04

**Authors:** Sadettin DEMİREL, Serdar ŞAHİNTÜRK, Naciye İŞBİL, Fadıl ÖZYENER

**Affiliations:** Department of Physiology, Faculty of Medicine, Bursa Uludağ University, Bursa, Turkey

**Keywords:** Irisin, rat thoracic aorta, vasodilation, protein kinase C

## Abstract

**Background/aim:**

Irisin, a newly identified exercise-derived myokine, has been found involved in a peripheral vasodilator effect. However, little is known regarding the potential vascular activity of irisin, and the mechanisms underlying its effects on vascular smooth muscle have not been fully elucidated. This study was aimed to investigate the effects of irisin on vascular smooth muscle contractility in rat thoracic aorta, and the hypothesis that protein kinase C (PKC) may have a role in these effects.

**Materials and methods:**

Isometric contraction-relaxation responses of thoracic aorta rings were measured with an isolated organ bath model. The steady contraction was induced with 10 μM phenylephrine (PHE), and then the concentration-dependent responses of irisin (0.001–1 μM) were examined. The time-matched vehicle control (double distilled water) group was also formed. To evaluate the role of PKC, endothelium-intact thoracic aorta rings were incubated with 150 nM bisindolylmaleimide I (BIM I) for 20 min before the addition of 10 μM PHE and irisin. Also, a vehicle control group was formed for dimethyl sulfoxide (DMSO).

**Results:**

Irisin exerted the vasorelaxant effects at concentrations of 0.01, 0.1, and 1 μM compared to the control group (p < 0.001). Besides, PKC inhibitor BIM I incubation significantly inhibited the relaxation responses induced by varying concentrations of irisin (p: 0.000 for 0.01 μM; p: 0.000 for 0.1 μM; p: 0.000 for 1 μM). However, DMSO, a solvent of BIM I, did not modulate the relaxant effects of irisin (p > 0.05).

**Conclusion:**

In conclusion, physiological findings were obtained regarding the functional relaxing effects of irisin in rat thoracic aorta. The findings demonstrated that irisin induces relaxation responses in endothelium-intact thoracic aorta rings in a concentration-dependent manner. Furthermore, this study is the first to report that irisin-induced relaxation responses are regulated probably via activating signaling pathways implicating PKC.

## 1. Introduction

Irisin, a newly identified hormone-containing 112 amino acids, which is secreted mainly by skeletal muscle cells during exercise, is a cleavage product of fibronectin type III domain-containing protein 5 (FNDC5) [1]. Irisin increases mitochondrial biogenesis and energy expenditure by activating the conversion of white adipocytes to brown adipocytes [1,2]. Circulating levels of irisin are associated with obesity, type II diabetes mellitus, insulin resistance, hypertension, and atherosclerosis. Recently, one of the agents predicted to be used in the prevention, follow-up, and treatment of these metabolic diseases and/or pathologies is the irisin molecule [3–5].

Vasoreactivity is of fundamental importance in various cardiovascular diseases such as hypertension, headache, and stroke [6]. Although some studies have demonstrated the peripheral vasodilator effect of irisin, little is known about the potential vascular activity of this molecule, and the mechanisms underlying its effects on vascular smooth muscle contraction-relaxation responses have not been fully elucidated yet. Besides, studies with this newly discovered adipomyokine have had several conflicting results. Therefore, more studies are needed to demonstrate the functional effects of irisin on vascular tissue and the mechanisms involved in these effects.

In the present study, it was aimed to investigate the effects of irisin on vascular smooth muscle contractility on rat thoracic aorta, a conducting artery, and the possible roles of protein kinase C (PKC) and dimethyl sulfoxide (DMSO), a solvent of PKC inhibitor bisindolylmaleimide I (BIM I).

## 2. Materials and methods

### 2.1. Ethics

This study was approved by the decision of Bursa Uludağ University Animal Experiments Local Ethics Committee dated 03/04/2019 and numbered 2019-04/10.

### 2.2. Test animals

In this study, male Wistar Albino rats (10–12 weeks of age), obtained from Bursa Uludağ University Experimental Animals Breeding Application and Research Center, were used. All experiments were performed according to the National Institutes of Health Guidelines on the Use of Laboratory Animals.

### 2.3. Chemicals and reagents used in experiments

Irisin, BIM I, phenylephrine hydrochloride (PHE), acetylcholine chloride (ACh), DMSO, and chemicals required to prepare Krebs solution used in the experiments were purchased from Sigma Aldrich (St Louis, MO, USA). Irisin, PHE, and ACh were dissolved in distilled water; BIM I was dissolved in DMSO.

### 2.4. Isolated organ bath experiments

#### 2.4.1. Preparation of aortic rings and mounting in organ baths

The animals used in the experiments were decapitated without anesthesia, taking care that they were not exposed to any stress factors. The thoracic cavity of the animals was opened rapidly and then the thoracic aorta was gently removed and placed in a Petri dish containing 0–4 °C Krebs solution. The Krebs solution contained: NaCl 119.0 mM, KCl 4.7 mM, MgSO_4_ 1.5 mM, KH_2_PO_4_ 1.2 mM, CaCl_2_ 2.5 mM, NaHCO_3_ 25.0 mM, and glucose 11.0 mM. The isolated vessels were cleared of adhering fat and connective tissue and then divided into 2–3 mm rings. Thoracic aorta rings were connected to the isometric force transducer (SS12LA force transducer, BIOPAC Systems, Inc. Aero Camino, USA) of the four-chamber organ bath (Commat Ltd., Ankara, Turkey) through steel wires as rapidly as possible to measure the isometric contractile responses. The temperature of the organ bath was kept constant at 37 °C (±0.5 °C) through a thermocirculator containing distilled water. The organ bath chambers were oxygenated with 95% O_2_-5% CO_2_ mixed gas during the experiment to maintain a pH of 7.4. Briefly, the isolated organ bath model was used as previously described [7].

#### 2.4.2. Vessel viability testing

Before each experiment, aortic rings were loaded with 2 g of resting tension and then stabilized for 60 min. The Krebs solution, containing the tissues, was refreshed every 15 min during the equilibration period. After this period, vessel viability and endothelial integrity were estimated by its stable and reproducible response to the addition of 10^−5^ M PHE and 10^−5^ M ACh. Those with >60% relaxation against ACh among the vascular segments were accepted as endothelium intact. Then, active, inhibitor, and vehicle substance applications were precisely performed into Krebs solution using an adjustable automatic pipette.

#### 2.4.3. Preliminary experiments

First, in preliminary experiments, PHE was added to isolated organ bath chambers in cumulative concentrations (0.0001–100 μM) after the equilibration period to determine the submaximal contraction dose. In preliminary experiments, it was also investigated that whether irisin, BIM I, and DMSO at the concentrations used in the study have significant effects on basal tone on rat thoracic aorta rings.

#### 2.4.4. Measurements of vascular reactivity in isolated thoracic aortas

Cumulative concentrations (0.001–1 μM) of irisin were used in all experimental groups. 10^−5^ M PHE was administrated to induce a steady contraction in the aortic rings, and then the concentration-dependent responses of irisin were examined to determine the vasodilative effect of irisin on the endothelium-intact thoracic aorta (control group). The time-matched vehicle control (double distilled water) group was also formed.

To evaluate the role of PKC, endothelium-intact thoracic aorta rings were incubated with 150 nM BIM I (PKC inhibitor) for 20 min before the addition of 10 μM PHE and irisin.

Following the incubation of inhibitor used for mechanism research, the responses developed depending on cumulative concentrations of irisin were compared with the control group, and then the differences between the relaxation amplitudes were calculated. Also, a “vehicle” control group was formed for DMSO, which was used as a solvent.

Eight aortic preparations were used for each of the experimental groups formed according to the active, inhibitor, or vehicle agents used in the study.

Contraction-relaxation responses were measured isometrically by force transducer on the MP36 recording system (BIOPAC Systems, Inc. Aero Camino, USA). The records obtained were evaluated in a computer environment with Biopac Student Lab 3.7.7 program (BIOPAC Systems, Inc. Aero Camino, USA).

### 2.5. Statistical analysis

The size of the difference between the groups was calculated by using Cohen’s d statistics for the comparison of the independent means and the effect size was determined as 0.30. While the power of the study was 0.80 and the alpha value was 0.05, the minimum sample size required was estimated at n = 5 per group. Power analysis was done under GPower 3.1. SPSS v.23.0 program (SPSS Inc., Chicago, IL, USA) was used for statistical analysis. Test of normality was determined by Shapiro Wilk test. The data were expressed as ‘Mean ± SD (Standard Deviation)’ for parametric data. The statistical significance between the two independent groups was analyzed using the independent sample t-test. One-way ANOVA was used for multiple comparisons. Dunnett’s T3 post hoc test was used to determine the differences between groups. For groups without the assumption of normality, Mann-Whitney U and Kruskal-Wallis with Dunn’s post-hoc test were used. P values of < 0.05 were considered statistically significant.

## 3. Results

In preliminary experiments, the submaximal contraction response was taken against 10 μM PHE. In all subsequent experiments, precontractions were induced with the addition of 10 μM PHE. Stable and reproducible contraction responses occurred in rat thoracic aorta rings with the submaximal concentration of PHE. In preliminary experiments, it was also determined that irisin, BIM I, and DMSO at the concentrations used in the study had no significant effects on basal tone in rat thoracic aorta rings.

### 3.1. Irisin induced vasodilation in rat thoracic aortas

**Figure 1:**
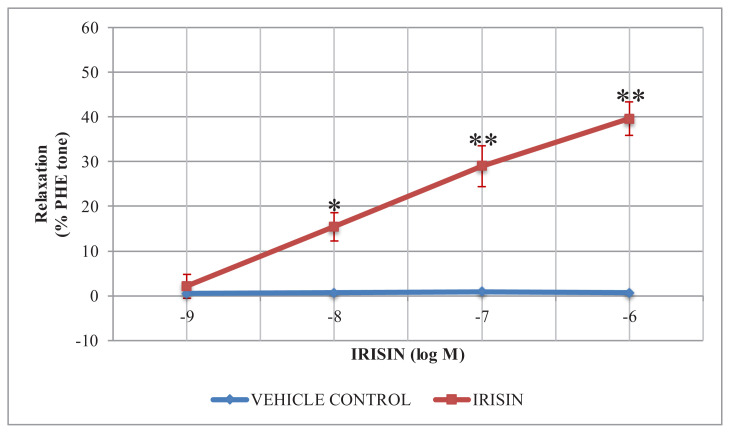
Concentration-dependent relaxant effects of irisin on PHE precontracted endothelium-intact rat thoracic aorta rings. The vehicle control group was treated with distilled water. The relaxant effects of irisin on thoracic aorta rings were calculated as a percentage of the contraction in response to 10 μM PHE. The data were expressed as the mean ± SD. (n = 8). *: p < 0.05 vs. control. **: p < 0.001 vs. control.

Cumulatively applied irisin induced concentration-dependent relaxation responses on endothelium-intact rat thoracic aorta rings precontracted with PHE. The relaxing effect of irisin was found to be significantly higher at concentrations of 0.01, 0.1, and 1 μM compared to the control group (double distilled water-treated rat thoracic aorta rings) (p: 0.589 for 0.001 μM; p: 0.014 for 0.01 μM; p: 0.000 for 0.1 μM; p: 0.000 for 1 μM) (Figure 1).

### 3.2. PKC was involved in the irisin-induced relaxation of rat thoracic aortas

**Figure 2:**
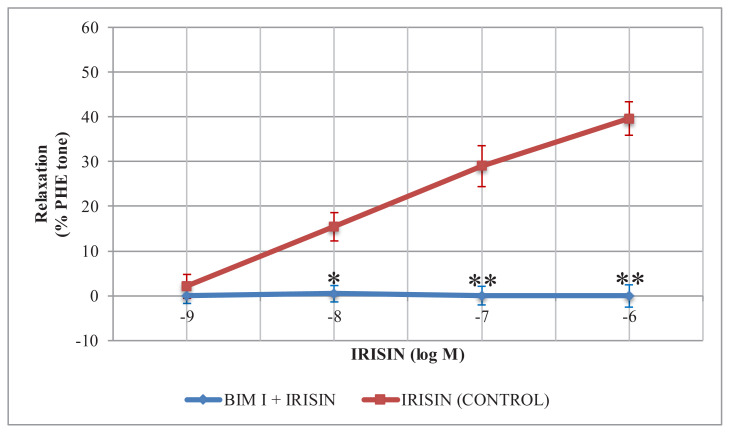
Effects of PKC inhibitor BIM I on irisin-induced relaxation in rat thoracic aorta. Relaxant effects of irisin on endothelium-intact thoracic aorta rings were measured in the absence (control) or presence of BIM I. The relaxant effects were calculated as a percentage of the contraction in response to 10 μM PHE. The data were expressed as the mean ± SD. (n = 8). *: p < 0.05 vs. control. **: p < 0.001 vs. control.

Incubation of tissues with PKC inhibitor BIM I (150 nM) for 20 min caused a significant change in the relaxation responses to irisin. BIM I incubation resulted in a statistically significant decrease in the relaxation responses of irisin applied at cumulative concentrations of 0.01, 0.1, and 1 μM (p: 0.039 for 0.01 μM; p: 0.000 for 0.1 μM; p: 0.000 for 1 μM). BIM I almost completely inhibited relaxation responses to irisin (Figure 2).

### 3.3. DMSO did not modulate the irisin-induced vasorelaxation effects

**Figure 3:**
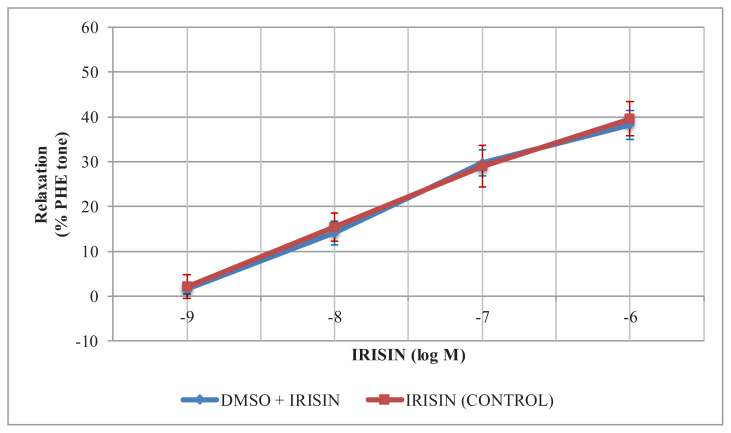
Effects of DMSO (at the solvent concentration of 50 μL) on irisin-induced relaxation in rat thoracic aorta. Relaxant effects of irisin on endothelium-intact thoracic aorta rings were measured in the absence (control) or presence of DMSO. The relaxant effects were calculated as a percentage of the contraction in response to 10 μM PHE. The data were expressed as the mean ± SD (n = 8).

Incubation of tissues with DMSO (50 μL) for 10 min did not cause a significant change in the relaxation responses to irisin. DMSO incubation at solvent concentration did not cause a remarkable change on any of the relaxation responses of irisin applied at cumulative concentrations of 0.01, 0.1, and 1 μM (p: 1.000 for 0.01 μM; p: 1.000 for 0.1 μM; p: 1.000 for 1 μM). DMSO did not significantly affect the relaxation responses to irisin (Figure 3) (Table).

## 4. Discussion

The aim of the present study was to investigate the functional relaxant effects of irisin on rat thoracic aorta and its underlying mechanisms. We have shown that irisin can significantly induce concentration-dependent relaxations in endothelium-intact rat thoracic aorta and the vasorelaxant effects of irisin were associated with PKC. The results suggest that irisin-induced relaxation responses are regulated probably via activating signaling pathways implicating PKC.

Despite great advances in medicine, proper treatment of arterial hypertension, diabetes mellitus, and chronic kidney disease remains a major challenge. Undiagnosed and untreated arterial hypertension or diabetes mellitus may lead to the development of chronic kidney disease and, consequently, to cardiovascular disorders [8].

The frequency of insulin resistance, obesity, and type II diabetes mellitus, and cardiovascular diseases such as hypertension and atherosclerosis due to these diseases has increased. Atherosclerosis, the main cause of coronary artery disease and stroke, remains the primary cause of global mortality [9,10].

Skeletal muscles have been defined as the endocrine organ in recent years [11]. Cytokines known as myokines, released from skeletal muscles during and/or immediately after exercise, mediate the beneficial effects of exercise on metabolism and the cardiovascular system [12,13].

Irisin, a newly described myokine, is increased with exercise and has the potential to play a preventive and/or therapeutic role in the management of the aforementioned chronic diseases [1,14,15–20]. In this context, it is considered that irisin may be an important therapeutic target in the prevention of the development of vascular pathologies and the treatment of atherosclerotic cardiovascular diseases. The precise functions of irisin related to the vascular tissue have not yet been clarified. However, few current studies have focused on the effects of irisin on vascular activity and the mechanisms involved.

Zhang et al. [15] have found that central administration of irisin increases blood pressure and cardiac contractility. In contrast, peripheral administration of irisin reduces blood pressure in both control and spontaneously hypertensive rats. Irisin dilates mesenteric artery rings through ATP-sensitive potassium channels. Han et al. [16] have provided the first evidence that irisin improves endothelial function in aortas of high-fat-diet-induced obese mice. The mechanism for this protective effect is related to the activation of the 5′ adenosine monophosphate-activated protein kinase (AMPK)-endothelial nitric oxide synthase (eNOS) signaling pathway. Jiang et al. [17] have investigated the effects of irisin on vascular activity and the mechanisms involved in mouse mesenteric arteries. They reported that irisin induced both endothelium-dependent and -independent relaxation in a dose-dependent manner. Endothelium-dependent relaxation is mediated by the nitric oxide (NO)-cyclic guanosine monophosphate (cGMP) dependent pathway, while endothelium-independent relaxation may be influenced by reducing Ca^2+^ influx through inhibiting voltage-dependent Ca^2+^ channels and intracellular Ca^2+^ release through both inositol-trisphosphate receptor (IP_3_R) and ryanodine receptor (RyR) channels. Fu et al. [18] have concluded that acute administration of irisin increases endothelium-dependent vasodilation and lowers the blood pressure of spontaneously hypertensive rats by amelioration of endothelial dysfunction of the mesenteric artery through the AMPK-protein kinase B (Akt)-eNOS-NO signaling pathway. Hou et al. [19] have shown that irisin improves the anticontractile properties of perivascular adipose tissue (PVAT) from the thoracic aorta in diet-induced obese mice. The mechanism for protective effects of irisin appears to be related to upregulation of the heme oxygenase-1/adiponectin axis in PVAT and browning of PVAT. Ye et al. [20] have stated that irisin-induced endothelium-dependent vasodilation is related to the stimulation of extracellular Ca^2+^ influx via transient receptor potential vanilloid subtype 4 (TRPV4) channels in rat mesenteric arteries.

PKC is a key regulatory enzyme involved in the transduction of signals in several cellular functions in vascular smooth muscle cells, including vascular smooth muscle contractility [21]. PKC regulates the contractility of vascular smooth muscle cells in various pathways, including changes in myofilaments Ca^2+^-sensitivity and regulation of intracellular free calcium concentration ([Ca^2+^]_i_, as well as by reducing endothelium-mediated vasodilation [22–24]. This enzyme is also involved in the regulation of the plasmalemma ion permeability in vascular smooth muscle cells. For instance, PKC has been shown to regulate the activity of K^+^ channels. PKC can influence vascular activity by physiological changes both in Ca^2+^ concentrations and membrane potential. The increased reactivity of the vascular wall is also associated with blunted endothelium-dependent responses due to reduced sensitivity to NO. Briefly, increased vascular smooth muscle cells myofilaments Ca^2+^ sensitivity and decreased sensitivity to NO can both be the result of a change in PKC activity [25]. The demonstration of a recent study that irisin suppresses oxidative/nitrative stresses by inhibiting PKC-β-related pathways in human umbilical vein endothelial cells cultured in high-glucose/high-fat [26] suggests that the vasodilator effect of irisin may also be due to the inhibition of signaling pathways implicating PKC. Although it is commonly accepted that PKC signaling inhibits eNOS activity, the distinct role of different PKC isoforms has not been studied so far. The PKC family comprises roughly 12 different isozymes that activate distinct downstream pathways. In this regard, activation of PKCα leads to phosphorylation of calponin, an actin-related regulatory protein in smooth muscle cells, resulting in vascular smooth muscle contraction; on the other hand, it can cause positive modulation of blood flow through the activation of eNOS in the endothelium [27,28].

In the present study, we investigated the role of PKC on the concentration-dependent relaxation responses of irisin. The vasorelaxant effects of irisin were significantly attenuated by pretreatment with PKC inhibitor BIM I, suggesting that signaling pathways implicating PKC may be associated with irisin-induced vasodilation. Thus, our results demonstrate for the first time that PKC stimulates irisin-induced vasodilation in rat thoracic aorta. However, PKC isoforms may have opposite effects on irisin-induced vasodilation. Therefore, further study is necessary to examine the hypothesis that irisin induces relaxation by enhancing the PKC-mediated nitric oxide synthesis in endothelium and/or inhibiting the PKC-regulated Ca sensitivity in vascular smooth muscle. Besides, further research using specific activators and inhibitors is required to clarify this effect.

Differences could be observed in the vasodilator activity of irisin on different vascular preparations obtained from various species. It is thought that this may be due to methodological differences such as the species from which the tissues were removed, the vascular tissues in which the experiments were performed (locations and diameters of vascular tissues), and/or the agents used to induce precontraction. Vasoreactivities of the arteries may be different from each other. It is known that the density and types of ion transport mechanisms and/or physiological/pharmacological receptors, as well as differences in substances released from the endothelium, could have an important role in changes in the responses of vascular smooth muscles to various agents [29,30]. For instance, the effects of hyperpolarizing factors increase inversely with vessel diameter [31]. Therefore, in the present study, the relaxing effects of irisin were investigated in thoracic aorta preparations isolated from Wistar Albino rats. Furthermore, the possible role of PKC regarding action mechanisms of irisin assessed in this study has not been performed on any species and/or vascular tissues before.

In this context, our results showed that 0.01, 0.1, and 1 μM concentrations of irisin have a vasodilatative effect on rat thoracic aorta, a conducting artery. Thus, we reported that irisin has relaxing effects on vascular smooth muscle contractility both at mean plasma concentration (0.1 μM) [18] and a wide range of concentrations, including the upper and lower levels of mean concentration (0.01–1 μM). Pretreatment with DMSO, a solvent of BIM I, did not modulate irisin-induced relaxation. It was further demonstrated that the irisin-induced vasorelaxation effects on endothelium-intact rat thoracic aorta were significantly abolished by pretreatment with PKC inhibitor BIM I. Thus, this study provides the first evidence that irisin-induced vasodilation is regulated probably via activating signaling pathways implicating PKC.

In conclusion, this study demonstrated that irisin induces concentration-dependent relaxation responses in endothelium-intact rat thoracic aorta rings. Furthermore, the present study is the first to report that the mechanism for this vasorelaxant effect is associated with the activity of PKC. These results suggest that irisin might be a useful agent in the treatment of abnormal vasoconstriction-related diseases such as hypertension and atherosclerosis, in addition to its key role in regulating energy metabolism, resulting in thermogenesis and weight loss, by increasing uncoupling protein-1 (UCP-1) expression in adipose tissue. This study further supports the beneficial effect of exercise on vascular function, depending on the release of irisin, an exercise hormone.

However, the findings presented in our study are preliminary. The precise roles of signaling pathways implicating PKC in irisin-mediated vasodilation need to be further elucidated in clinically relevant animal models. Besides, more detailed mechanism studies and in vivo studies are necessary to establish the efficacy of irisin in the management and treatment of cardiovascular diseases.

## Figures and Tables

**Table:** Descriptive statistics for each group. Outliers have been corrected.

	Concentration (log M)	Mean	Median	Std. Deviation	Test of Normality	
					Shapiro-Wilk	
					Statistic	p
**VEHICLE CONTROL (n = 8)**	−**9**	0.50	0.00	0.00	0.724	0.004
	−**8**	0.62	0.00	0.00	0.693	0.002
	−**7**	0.87	0.50	0.01	0.809	0.036
	−**6**	0.62	0.00	0.00	0.693	0.002
**IRISIN (n = 8)**	−**9**	2.12	1.50	2.66	0.945	0.662
	−**8**	15.50	15.00	3.16	0.957	0.785
	−**7**	29.00	29.50	4.62	0.943	0.644
	−**6**	39.62	39.50	3.77	0.954	0.747
**BIM-I (n = 8)**	−**9**	0.00	0.50	1.71	0.929	0.503
	−**8**	0.50	2.50	1.80	0.786	0.020
	−**7**	0.00	0.50	2.06	0.911	0.361
	−**6**	0.00	0.50	2.47	0.894	0.255
**DMSO (n = 8)**	−**9**	1.62	2.00	1.08	0.875	0.168
	−**8**	14.12	13.50	2.70	0.913	0.378
	−**7**	29.75	30.00	2.85	0.952	0.727
	−**6**	38.25	39.50	3.26	0.913	0.377

## References

[1] Boström P Wu J Jedrychowski MP Korde A Ye L A PGC1-α-dependent myokine that drives brown-fat-like development of white fat and thermogenesis Nature 2012 481 463 468 10.1038/nature10777 22237023 PMC3522098

[2] Sanchis-Gomar F Lippi G Mayero S Perez-Quilis C García-Giménez JL Irisin: a new potential hormonal target for the treatment of obesity and type 2 diabetes Journal of Diabetes 2012 4 196 10.1111/j.1753-0407.2012.00194.x 22372821

[3] Polyzos SA Anastasilakis AD Efstathiadou ZA Makras P Perakakis N Irisin in metabolic diseases Endocrine 2018 59 260 274 10.1007/s12020-017-1476-1 29170905

[4] Korta P Pocheć E Mazur-Biały A Irisin as a multifunctional protein: implications for health and certain diseases Medicina (Kaunas) 2019 55 485 10.3390/medicina55080485 31443222 PMC6722973

[5] Ma C Ding H Deng Y Liu H Xiong X Irisin: a new code uncover the relationship of skeletal muscle and cardiovascular health during exercise Frontiers in Physiology 2021 12 620608 10.3389/fphys.2021.620608 33597894 PMC7882619

[6] Brunner H Cockcroft JR Deanfield J Donald A Ferrannini E Endothelial function and dysfunction. Part II: association with cardiovascular risk factors and diseases. A statement by the Working Group on Endothelins and Endothelial Factors of the European Society of Hypertension Journal of Hypertension 2005 23 233 246 10.1097/00004872-200502000-00001 15662207

[7] Jespersen B Tykocki NR Watts SW Cobbett PJ Measurement of smooth muscle function in the isolated tissue bath-applications to pharmacology research Journal of Visualized Experiments 2015 19 52324 10.3791/52324 PMC4354551 25650585

[8] Maciorkowska M Musiałowska D Małyszko J Adropin and irisin in arterial hypertension, diabetes mellitus and chronic kidney disease Advances in Clinical and Experimental Medicine 2019 28 1571 1575 10.17219/acem/104551 31756066

[9] Byun K Lee S The potential role of irisin in vascular function and atherosclerosis: a review International Journal of Molecular Sciences 2020 21 7184 10.3390/ijms21197184 33003348 PMC7583934

[10] Voutyritsa E Kyriakos G Patsouras A Damaskos C Garmpi A Experimental agents for the treatment of atherosclerosis: new directions Journal of Experimental Pharmacology 2021 13 161 179 10.2147/JEP.S265642 33633471 PMC7901406

[11] Pedersen BK Akerström TC Nielsen AR Fischer CP Role of myokines in exercise and metabolism Journal of Applied Physiology 2007 103 1093 1098 10.1152/japplphysiol.00080.2007 17347387

[12] Pedersen BK Febbraio MA Muscles, exercise and obesity: skeletal muscle as a secretory organ Nature Reviews Endocrinology 2012 8 457 465 10.1038/nrendo.2012.49 22473333

[13] Strasser B Physical activity in obesity and metabolic syndrome Annals of the New York Academy of Sciences 2013 1281 141 159 10.1111/j.1749-6632.2012.06785.x 23167451 PMC3715111

[14] Demirel S Şahintürk S Özyener F Irisin and its effects on vascular contractility Medical Journal of Suleyman Demirel University 2021 28 163 170 10.17343/sdutfd.718412

[15] Zhang W Chang L Zhang C Zhang R Li Z Central and peripheral irisin differentially regulate blood pressure Cardiovascular Drugs and Therapy 2015 29 121 127 10.1007/s10557-015-6580-y 25820670 PMC4431576

[16] Han F Zhang S Hou N Wang D Sun X Irisin improves endothelial function in obese mice through the AMPK-eNOS pathway American Journal of Physiology-Heart and Circulatory 2015 309 H1501 H1508 10.1152/ajpheart.00443.2015 26371167

[17] Jiang M Wan F Wang F Wu Q Irisin relaxes mouse mesenteric arteries through endothelium-dependent and endothelium-independent mechanisms Biochemical and Biophysical Research Communications 2015 468 832 836 10.1016/j.bbrc.2015.11.040 26582714

[18] Fu J Han Y Wang J Liu Y Zheng S Irisin lowers blood pressure by improvement of endothelial dysfunction via AMPK-Akt-eNOS-NO pathway in the spontaneously hypertensive rat Journal of the American Heart Association 2016 5 e003433 10.1161/JAHA.116.003433 27912206 PMC5210324

[19] Hou N Liu Y Han F Wang D Hou X Irisin improves perivascular adipose tissue dysfunction via regulation of the heme oxygenase-1/adiponectin axis in diet-induced obese mice Journal of Molecular and Cellular Cardiology 2016 99 188 196 10.1016/j.yjmcc.2016.09.005 27638193

[20] Ye L Xu M Hu M Zhang H Tan X TRPV4 is involved in irisin-induced endothelium-dependent vasodilation Biochemical and Biophysical Research Communications 2017 495 41 45 10.1016/j.bbrc.2017.10.160 29097199

[21] Cosentino-Gomes D Rocco-Machado N Meyer-Fernandes JR Cell signaling through protein kinase C oxidation and activation International Journal of Molecular Sciences 2012 13 10697 10721 10.3390/ijms130910697 23109817 PMC3472709

[22] Somlyo AP Somlyo AV Ca2+ sensitivity of smooth muscle and nonmuscle myosin II: modulated by G proteins, kinases, and myosin phosphatase Physiological Reviews 2003 83 1325 1258 10.1152/physrev.00023.2003 14506307

[23] Massett MP Ungvari Z Csiszar A Kaley G Koller A Different roles of PKC and MAP kinases in arteriolar constrictions to pressure and agonists American Journal of Physiology-Heart and Circulatory 2002 283 H2282 H2287 10.1152/ajpheart.00544.2002 12427592

[24] Korzick DH Laughlin MH Bowles DK Alterations in PKC signalling underlie enhanced myogenic tone in exercise-trained porcine coronary resistance arteries Journal of Applied Physiology 2004 96 1425 1432 10.1152/japplphysiol.01077.2003 14672961

[25] Soloviev AI Kizub IV Mechanisms of vascular dysfunction evoked by ionizing radiation and possible targets for its pharmacological correction Biochemical Pharmacology 2018 159 121 139 10.1016/j.bcp.2018.11.019 30508525

[26] Zhu D Wang H Zhang J Zhang X Xin C Irisin improves endothelial function in type 2 diabetes through reducing oxidative/nitrative stresses Journal of Molecular and Cellular Cardiology 2015 87 138 147 10.1016/j.yjmcc.2015.07.015 26225842

[27] Partovian C Zhuang Z Moodie K Lin M Ouchi N PKCalpha activates eNOS and increases arterial blood flow in vivo Circulation Research 2005 97 482 487 10.1161/01.RES.0000179775.04114.45 16081872

[28] Wang Y Zhou H Wu B Zhou Q Cui D Protein kinase C isoforms distinctly regulate propofol-induced endothelium-dependent and endothelium-independent vasodilation Journal of Cardiovascular Pharmacology 2015 66 276 284 10.1097/FJC.0000000000000275 25970840

[29] Clark SG Fuchs LC Role of nitric oxide and Ca^++^-dependent K^+^ channels in mediating heterogeneous microvascular responses to acetylcholine in different vascular beds Journal of Pharmacology and Experimental Therapeutics 1997 282 1473 1479 9316861

[30] Mulvany MJ Aalkjaer C Structure and function of small arteries Physiological Reviews 1990 70 921 961 10.1152/physrev.1990.70.4.921 2217559

[31] de Wit C Wölfle SE EDHF and gap junctions: important regulators of vascular tone within the microcirculation Current Pharmaceutical Biotechnology 2007 8 11 25 10.2174/138920107779941462 17311549

